# POEMS (Polyneuropathy, Organomegaly, Endocrinopathy, Monoclonal Protein, and Skin Changes): A Case Report of a Rare Paraneoplastic Syndrome

**DOI:** 10.7759/cureus.24980

**Published:** 2022-05-13

**Authors:** Anuradha Sakhuja, Dhan B Shrestha, Wasey Ali Yadullahi Mir, Suman Gaire, Mohammed Kassem

**Affiliations:** 1 Department of Internal Medicine, Mount Sinai Hospital, Chicago, USA; 2 Department of Emergency Medicine, Palpa Hospital, Palpa, NPL; 3 Department of Hematology and Oncology, Mount Sinai Hospital, Chicago, USA

**Keywords:** hyperpigmentation, osteosclerosis, paraproteinemias, polyneuropathy, poems syndrome

## Abstract

POEMS (polyneuropathy, organomegaly, endocrinopathy, monoclonal protein, and skin changes) syndrome is a multisystem disorder. Peripheral neuropathy and monoclonal plasma cell disorder are the most common manifestations of POEMS. Although osteosclerotic or mixed sclerotic-lytic lesions are typical, osteolytic lesions are rarely encountered. We present a case of a 39-year-old male with a history of multiple endocrine disorders who presented with paresthesia, edema, and hyperpigmentation and was eventually diagnosed with POEMS syndrome. Patients with unexplained neuropathy should be evaluated for POEMS syndrome, especially when it is associated with other findings like endocrinopathy, organomegaly, skin changes, or edema.

## Introduction

POEMS is an acronym for the prominent features of the syndrome that the term represents: polyneuropathy, organomegaly, endocrinopathy, monoclonal protein, and skin changes. The acronym was coined to describe the disorder based on two case reports in 1980 [1]. Over time, many other disease features have been observed, including osteosclerotic bone lesion, Castleman disease, edema, and thrombocytosis [2]. It is a rare multisystem disorder associated with underlying plasma cell disorder. It is often difficult to diagnose the condition because of its rarity and multisystem presentation. However, once diagnosed, the disease responds well to treatment. The incidence rate of POEMS syndrome has not been estimated because of its rarity.

In this report, we present a case of a 39-year-old Hispanic male suffering from multiple endocrine abnormalities who later developed paresthesia, edema, and skin changes and was eventually diagnosed with POEMS syndrome.

## Case presentation

**Figure 1:**
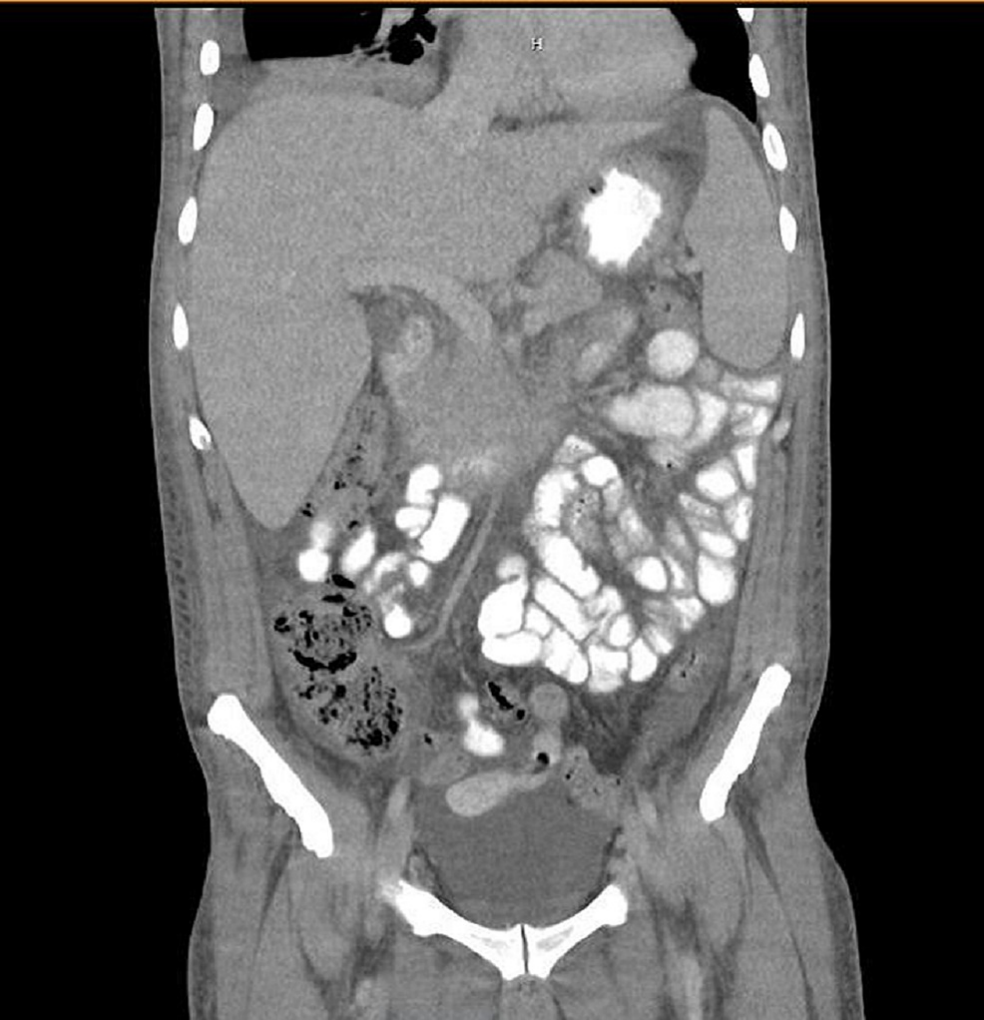
CT scan of abdomen and pelvis showing pleural effusion and hepatosplenomegaly CT: computed tomography

**Figure 2:**
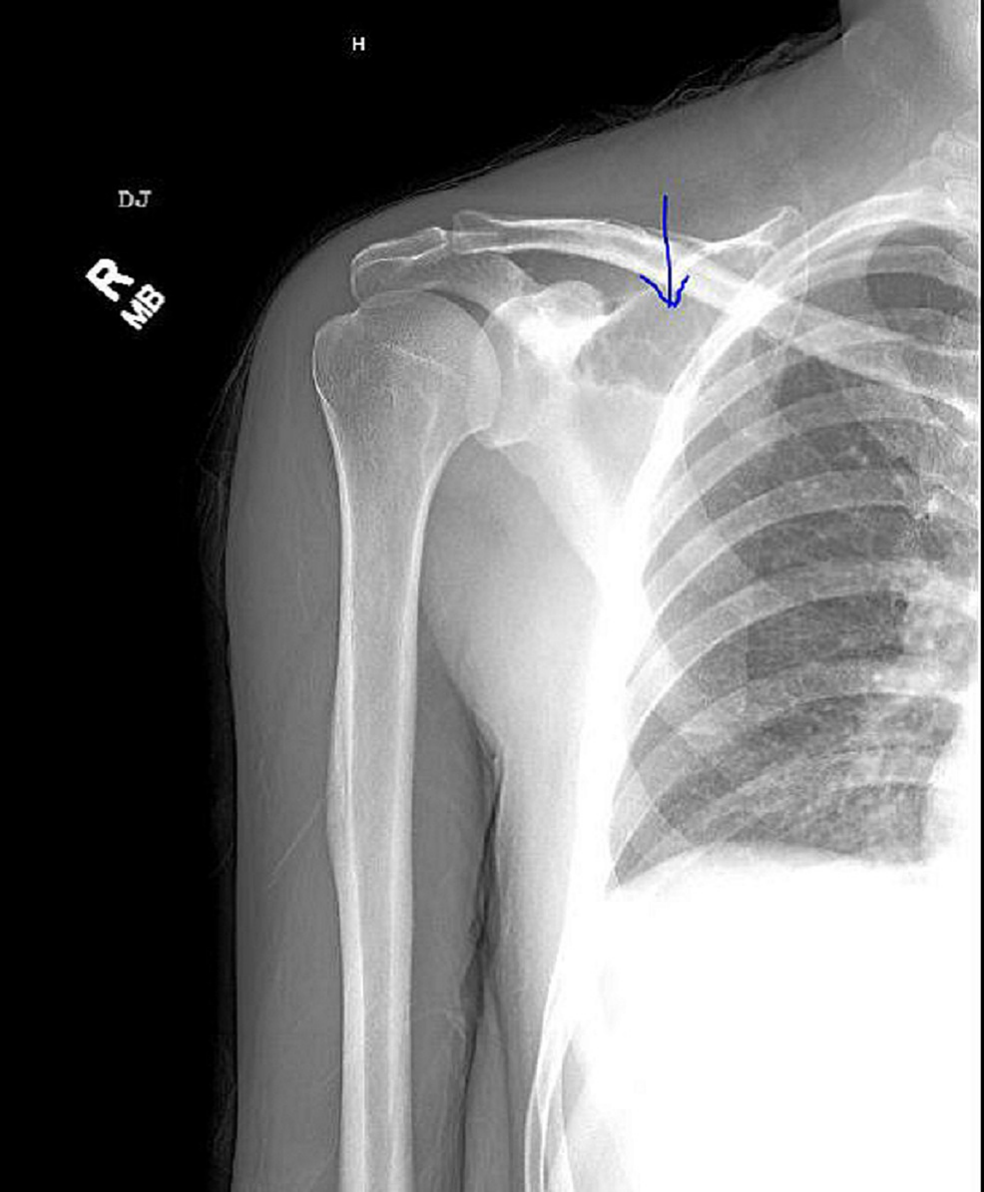
X-ray of the right shoulder The blue arrow shows a 4.9 cm x 8.6-cm lobulated osteolytic lesion in the superior medial right scapula

**Figure 3:**
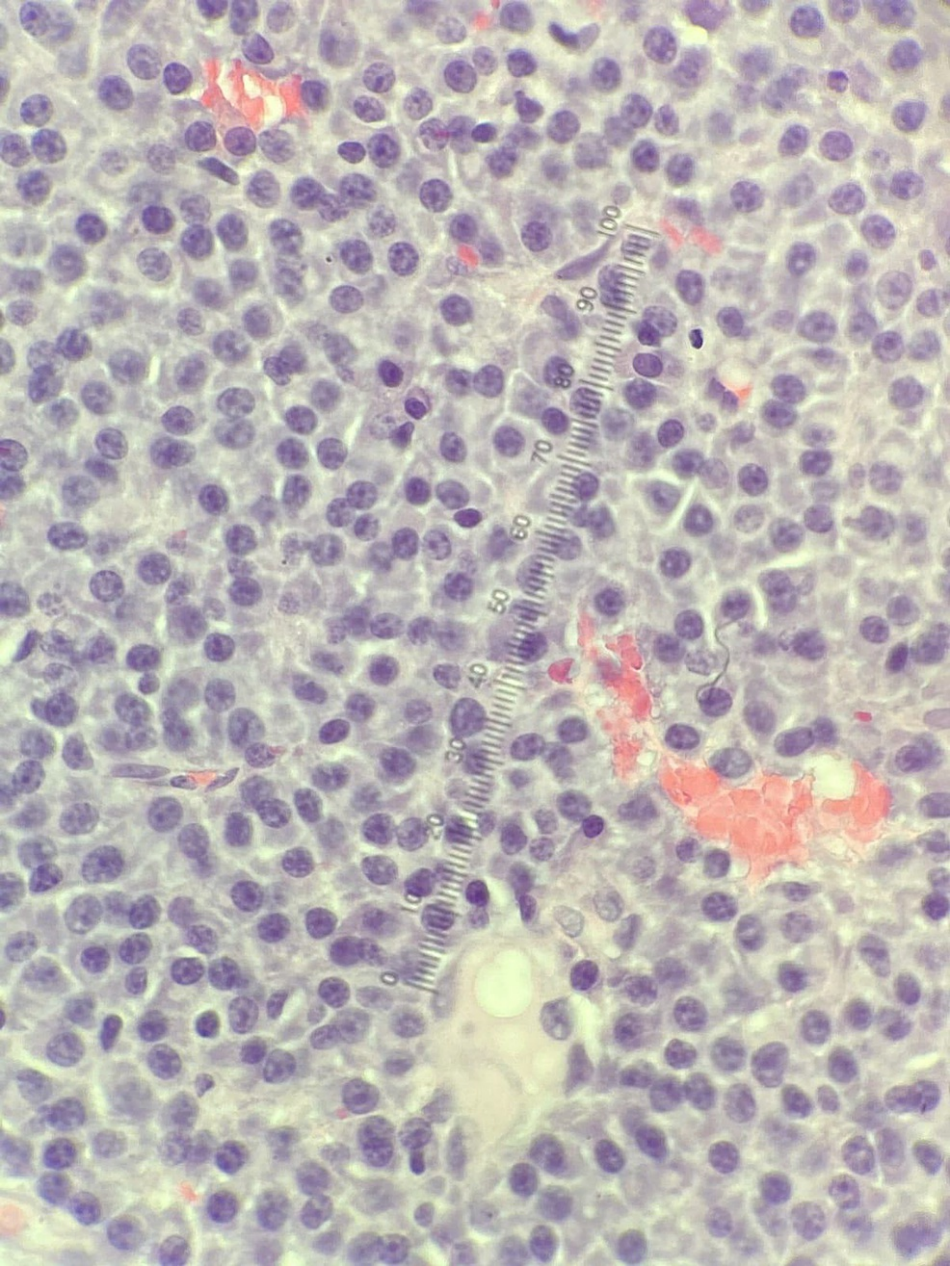
Pathological smear showing diffuse sheets of CD138-positive plasma cells

A 39-year-old male presented to the Hematology clinic with a three-year history of generalized swelling, skin erythematous vascular papular lesions of his right chest/shoulder and left arms, paresthesias of bilateral feet, right shoulder pain, and hypertrichosis. He had been diagnosed with adrenal insufficiency and hypothyroidism and managed with hydrocortisone and levothyroxine. He also had anasarca, proteinuria, generalized fatigue, unintentional 30-pound weight loss over the last three years, night sweats, and skin hyperpigmentation. Initial laboratory reports suggested polycythemia with a hemoglobin level of 18 g/dL; autoimmune and paraproteinemia workup was negative. Electrophoresis was concerning for IgG lambda monoclonal gammopathy on immunofixation. Bone marrow biopsy was performed, which revealed normocellular marrow with trilineage hematopoiesis, and mildly increased plasma cells around 10% with no monoclonality. CT scan of the chest, abdomen, and pelvis showed diffuse anasarca with bilateral pleural effusion, hepatosplenomegaly (Figure 1), and slightly enlarged axillary, mediastinal, and iliac chain lymph nodes. The skeletal survey showed a 4.9 x 8.6-cm lobulated osteolytic lesion in the superior medial right scapula (Figure 2). The patient underwent an interventional radiology (IR)-guided biopsy revealing an extraosseous infiltrative plasmacytoma. The core biopsy showed >90% cells consisting of diffuse sheets of CD138-positive plasma cells (plasma cell neoplasm) (Figure 3). He was started on radiation therapy for scapular plasmacytoma and then treated with zoledronic acid.

The patient's skin lesions on the right upper chest skin were shave-biopsied, and they turned out to be papillary hemangiomas. Based on his signs and symptoms, he met the major and minor criteria for the diagnosis of POEMS syndrome. This diagnosis was further supported by an elevated vascular endothelial growth factor (VEGF) level.

The patient has followed up with multiple specialties, including Hematology/Oncology, Endocrinology, and Dermatology regularly since his first presentation, and it has already been three years. His skin lesions are now stable; endocrinopathies are managed by levothyroxine and hydrocortisone, and the left scapular pain has resolved. Neuropathy has improved and is controlled on duloxetine. The scapular lesion has decreased in size drastically on repeat scans, and VEGF levels have decreased from the initial diagnosis value of 288 pg/ml to 56 pg/ml during the one-and-half-year follow-up course.

## Discussion

In this study, we discuss an infrequent multisystem disorder known as POEMS syndrome. The diagnosis of POEMS syndrome involves a three-step approach that includes mandatory, major, and minor criteria for diagnosis [3]. Peripheral neuropathy and a monoclonal plasma cell disorder (almost always lambda light chain type) are mandatory for diagnosis. At least one factor is required to be present from major and minor criteria. The major criteria include elevated VEGF levels, Castleman disease, and sclerotic bone lesions. Minor criteria include organomegaly, endocrinopathy, extravascular volume overload, skin changes, thrombocytopenia/polycythemia, and papilledema [3]. Our patient met both mandatory criteria and most of the major and minor criteria. Further investigation revealed an osteolytic lesion in the right scapular area. All the above symptoms, along with elevated VEGF levels, confirmed the diagnosis of POEMS syndrome.

Peripheral neuropathy is a major finding in POEMS syndrome and is seen in almost 100% of the cases [4]. In POEMS syndrome, patients suffer from subacute-onset demyelinating neuropathy that starts with sensory symptoms and progresses to motor symptoms with disease progression. Symptoms usually include paraesthesia, tingling, and, eventually, debilitating motor symptoms. Although a case without polyneuropathy has been reported, it seems to have been a rare occurrence [5]. Our patient suffered from multiple endocrinopathies and peripheral neuropathy symptoms, edema, and dermatological symptoms. This prompted the evaluation for POEMS syndrome.

The osteosclerotic bone lesion is also a significant finding in POEMS syndrome. Hence, the disease was called osteosclerotic myeloma in the past. While osteosclerotic lesions, including mixed osteosclerotic and osteolytic lesions, are seen in up to 97% of patients with POEMS syndrome, pure osteolytic presentations are rare [2]. Osteolytic lesions have been reported in about 2-14% of patients with POEMS syndrome [2,6]. Our patient presented with a single osteolytic bone lesion in the scapula.

The primary cause leading to multisystem involvement in POEMS syndrome is unknown. However, it has been suggested that overproduction of VEGF and proinflammatory cytokines including interleukin-1 beta (IL-1ß), tumor necrosis factor-alpha (TNF-alpha), and interleukin-6 (IL-6) may play a major role in the pathogenesis [7,8]. In addition, elevated VEGF increases the microvascular permeability of nerves causing endoneurial edema and increasing access of complements and thrombin to nerve cells, thereby causing nerve damage [9]. Therefore, the plasma VEGF levels are used both to diagnose the disease and monitor the disease process as the decreased levels also correlate with clinical improvement [10].

There is no established treatment protocol for POEMS syndrome, as randomized controlled trials for the treatment of POEMS have not been conducted. Hence, the management is guided by case series and observational studies. The treatment options include radiotherapy, chemotherapy, immunomodulation, and hematopoietic stem cell transplantation [11]. Radiotherapy is preferred for patients with no bone marrow involvement and three or fewer isolated osseous lesions [4,12]. Hence, our patient was treated with radiotherapy. The patient's condition has improved with the treatment.

## Conclusions

POEMS syndrome is a rare disorder that encompasses various symptoms involving multiple systems and is difficult to diagnose. Patients with unexplained neuropathy should be evaluated for POEMS syndrome, especially if it is associated with other suggestive features like endocrinopathy, edema, and osseous lesions. Although patients often present with osteosclerotic lesions, isolated osteolytic lesions can be associated with POEMS syndrome.
